# Maternal unwanted and intrusive thoughts of infant-related harm, obsessive-compulsive disorder and depression in the perinatal period: study protocol

**DOI:** 10.1186/s12888-019-2067-x

**Published:** 2019-03-21

**Authors:** Fanie Collardeau, Bryony Corbyn, John Abramowitz, Patricia A. Janssen, Sheila Woody, Nichole Fairbrother

**Affiliations:** 1 Department of Psychology, University of Victoria, Victoria, Canada; 2 Department of Psychiatry, University of British Columbia, Vancouver, Canada; 3 Department of Psychology, University of North Carolina at Chapel Hill, Chapel Hill, USA; 4 School of Population and Public Health, University of British Columbia, Vancouver, Canada; 5 Department of Psychology, University of British Columbia, Vancouver, Canada

**Keywords:** Epidemiology, Perinatal, Depression, OCD, Intrusive thoughts, Infant harm

## Abstract

**Background:**

Unwanted, intrusive thoughts of harm-related to the infant are reported by the vast majority of new mothers, with half of all new mothers reporting unwanted, intrusive thoughts of harming their infant on purpose. Thoughts of intentional harm, in particular, are distressing to women, their partners and the people who care for them. While maternal, unwanted and intrusive thoughts of infant-related harm are known to be associated with obsessive compulsive disorder (OCD) and depression, preliminary evidence suggests that they are not associated with an increased risk of harm to infants. Perinatal care providers and policy makers, as well as new mothers and their partners require evidence-based information in order to respond appropriately to these types of thoughts. The purpose of this research is to address important gaps regarding the (a) prevalence and characteristics of intrusive, unwanted thoughts of baby-related harm, (b) their association (or lack thereof) with child abuse, and (c) the prevalence and course of obsessive-compulsive disorder and depression in the perinatal period.

**Methods:**

Participant were 763 English-speaking women and recruited during pregnancy. In this province-wide study in British Columbia, participants were recruited proportionally from hospitals, city centers and rural communities between January 23, 2014 and September 09, 2016. Participants were administered online questionnaires and diagnostic interviews over the phone at 33-weeks gestation, 7-weeks postpartum and 4-months postpartum. The study assessed intrusive and unwanted thoughts of harm related to the infant, obsessive-compulsive disorder (OCD) and major depressive episode (MDE) disorders and symptomatology, sleep, medical outcomes, parenting attitudes, and infant abuse.

**Discussion:**

There is a scarcity of literature concerning maternal unwanted, intrusive, postpartum thoughts of infant-related harm and their relationship to child harming behaviors, OCD and depression. This longitudinal cohort study was designed to build on the existing research base to ensure that policy developers, child protection workers and health-care providers have the guidance they need to respond appropriately to the disclosure of infant-related harm thoughts. Thus, its main goals will be to investigate whether intrusive postpartum thoughts of infant-related harm are a risk factor for child abuse or the development of OCD.

## Background

Between 70 and 100% of new mothers report unwanted, intrusive thoughts of infant-related harm with as many as half of all new mothers reporting unwanted, intrusive thoughts of harming their infant on purpose [1, 2, 3]. These thoughts may include, for example, ideas of suffocation and sudden infant death syndrome (81.4–90%), accidents (83.7–92%), contamination (53.5–59%), or intentional harm (32.6–46%) [2, 3]. Such thoughts usually peak in frequency during the first few weeks postpartum [4]. Although women vary in how distressing they experience postpartum intrusive thoughts of infant-related harm to be, thoughts of intentional harm (i.e., harming the infant on purpose) have been found to be particularly upsetting [1, 5]. A key concern in this area of work is the real possibility that unwanted, postpartum intrusions of infant-related harm represent a risk factor for infant-related harming behaviours (i.e., maternal aggression towards the infant). If unwanted, intrusive thoughts of harm are predictors of harming behaviors, then acting to protect the infant is appropriate and necessary. Conversely, if those unwanted, intrusive thoughts of infant-related harm are actually a common and normative postpartum experience which may predispose to mental health difficulties (e.g., obsessive compulsive disorder; OCD) among vulnerable women, taking dramatic steps (e.g., intense monitoring of the mother or removal of the infant) is unnecessary and may in fact be harmful.

Preliminary evidence suggests that maternal, unwanted, postpartum, intrusions of infant-related harm do not predict harming behaviors toward the infant [1, 6] and closely resemble the unwanted, intrusive thoughts and images and impulses experienced by 80 to 99% of the general population [7–12]. These types of very common thoughts have been shown to predispose to the development and exacerbation of OCD in vulnerable individuals. Further, unwanted, intrusive thoughts, images and impulses reported by the general population, postpartum intrusive ideation are related to one’s on-going concerns [13, 14] and appear to be more frequent in the context of stressful emotion situations and negative emotional states [15–17]. They do not, however, reflect the person’s actual wishes or intentions. Unsurprisingly, when new mothers’ experience unwanted, intrusive thoughts, the content of the thoughts is frequently related to the infant. Cognitive-behavioural theory posits that normally occurring, albeit unwanted, intrusive thoughts may develop into clinical obsessions and in turn lead to the development of OCD if the person experiencing the thoughts makes catastrophically negative appraisals of the meaning of the occurrence and/or the content of the thoughts [14, 18–23]. Preliminary data in this area suggests that this is true for postpartum unwanted and intrusive thoughts of infant-related harm also [5, 23].

OCD is an anxiety-related disorder characterized by obsessions and/or compulsions. Obsessions are recurrent, unwanted and distressing thoughts, images, or impulses [24]. Normal intrusive thoughts differ from clinical obsessions by virtue of the time they take and the distress and impairment they cause [4]. Compulsions are repetitive mental or behavioral acts that a person engages in, and are often undertaken to decrease the distress associated with obsessions [24]. There is now strong evidence that the perinatal period is a time of increased risk for the development and exacerbation of OCD symptoms [14], with prevalence estimates in pregnancy and the post-partum period usually exceeding the ones for adult women in the general population [14, 25, 26]. In addition, OCD symptoms can sometimes be compounded by or lead to additional mental health difficulties, including depression [27–30].

Postnatal OCD has potentially negative implications for mothering, marital functioning, infant development and social support [26, 31–34]. Furthermore, stress and anxiety in pregnancy are associated with impaired fetal and infant development, maternal distress, and negative cognitive and temperamental outcomes for the infant [26, 31, 32, 35–39]. Currently, there is no clear evidence upon which to base the best response when a woman discloses that she is experiencing unwanted, intrusive thoughts of infant-related harm. This study aims to fill some of these gaps in knowledge and provide evidence upon which women, their partners, maternity care providers and policy makers can base decisions regarding the most appropriate course of action to take when a new mother discloses unwanted, intrusive thoughts of infant-related harm. Thus, the main objective of the current research is to further investigate the implications of unwanted, intrusive thoughts of infant-related harm and to answer two key questions: are unwanted, intrusive thoughts of infant-related harm predictors of harming behaviors toward the infant? Are unwanted, intrusive thoughts of infant-related harm predictors of postpartum OCD? If unwanted, intrusive thoughts of infant-related harm predicts or plays a role in the maintenance of postpartum OCD, identifying women who struggle with those thoughts and providing them with adequate information or treatment will decrease their OCD symptoms. This study is designed to expand the current literature on three broad areas of research: (a) perinatal intrusive thoughts of infant-related harm (b) maternal aggression towards the infant (c) perinatal OCD and depression epidemiology.

## Study objectives

### Perinatal intrusive thoughts of infant-related harm

Our objectives in this area were to determine:

- the prevalence of maternal, unwanted, intrusive thoughts of infant-related harm at 7-weeks and 4-months postpartum,
- the course of maternal, unwanted, intrusive thoughts of infant-related harm over the first 4-months postpartum,
- the content and characteristics of new mothers’ unwanted, intrusive thoughts of harm, and
- the role of maternal beliefs about maternal unwanted and intrusive thoughts of infant-related harm in predicting postpartum OCD symptoms.

### Maternal aggression toward the infant

With respect to maternal aggression towards the infant, we sought to determine if:

- maternal intrusive and unwanted thoughts of infant-related harm predict maternal aggression toward the infant, and
- parenting attitudes and beliefs, but not maternal intrusive, unwanted thoughts of infant-related harm, predict maternal aggression toward the infant.

### Perinatal OCD and major depressive episode (MDE)

With respect to OCD we aimed to determine:

- the prevalence of OCD in the perinatal period (i.e., the third trimester of pregnancy and at 7-weeks and 4-months postpartum),
- the course of OCD symptoms over the perinatal period, and
- the role of postpartum social support, maternal sleep, and infant temperament in predicting OCD symptoms, mood, and intrusive thoughts of infant-related harm at 4-months postpartum.

With respect to depression, we aimed to determine:

- the prevalence and course of MDE and depressive symptoms over the perinatal period.

## Methods

### Study design

This is a prospective, cohort study.

### Inclusion/exclusion criteria

All English-speaking pregnant women living in the Province of British Columbia (BC) during the study recruitment time frame were eligible to participate. British Columbia, Canada was selected as the geographical boundary for the research. Although all eligible participants were encouraged to complete all time points, women who had not completed a questionnaire and/or interview at earlier time points were nevertheless eligible to complete the questionnaire and interview at later time points. Women who were below 19 years of age were excluded.

### Recruitment

In order to achieve a sampling frame that was representative of the population of birthing women in BC, we employed a range of recruitment strategies which encompassed hospital-based recruitment (85.3%, *n* = 898), community-based recruitment (13.3%, 140), and rurally focused approaches (1.4%, *n* = 15). The Statistics Canada definition of rural was used for this research. Participants were recruited from January 23, 2014 until September 09, 2016. Direct recruitment methods (i.e., approaching women as they wait for their routine antenatal appointments) were used at several hospitals including BC Children and Women’s, St Paul’s Hospital, Royal Columbian Hospital, Burnaby Hospital, Surrey Memorial Hospital, Lion’s Gate Hospital, Langley Hospital and Victoria General. Indirect recruitment methods were used at different sites including Abbotsford Regional Hospital, UBC Family Practice Clinic, and private clinics and prenatal centers in Abbotsford, greater Vancouver, Kelowna, Vancouver Island, and the Fraser Valley. Community and rural recruitment involved providing introductory letters and study pamphlets to midwives, family physicians and obstetricians, and placing posters and pamphlets at trade shows, community events and in prenatal education classes or community sites throughout BC (e.g., community centers).

### Sample size estimation

Our earlier work indicated that we could expect approximately 50% of our sample to report thoughts of intentional harm, and 5% of our sample to report some form of physical aggression towards their infant [1]. As a result, to detect an absolute difference of ≥5% with 94% power in the rate of maternal aggression between women who report thoughts of intentional harm and women who do not (2.5% vs. 7.5%), a sample size of 1000 new mothers would be required [40, 41]. All other study questions and hypotheses (including hypotheses pertaining to OCD prevalence) have smaller sample size requirements.

### Representativeness

To maximize the representativeness of our sample, we engaged in the following approaches:

- we utilized the recruitment strategies outlined above, in an effort to ensure recruitment across all geographical and socioeconomic regions of BC,
- we recruited proportionally from each of the participating hospitals, and
- we plan to use data weighting, based on data provided to us by Perinatal Services BC (2018), for the following variables: birth location (hospital name or home delivery), parity, maternal age, care provider type, mode of delivery to further align our sample with the population of birthing women in BC.

Provincial data (for the purposes of data weighting) will be provided by Perinatal Services BC. According to information provided by the British Columbia Perinatal Data Registry, during the period of data collection (February 09, 2014 to February 14, 2017), 133,088 women gave birth in the province [42]. Sixty-nine percent (69.2%) of these births took place at locations targeted for recruitment by the present study. In British Columbia 3.3% of the births took place at home. Fewer than half (46.2%) of the women in BC had never given birth before. Most were between 30 and 34 years old (37.6%), 25–29 years-old (25.8%) and 35–39 years-old (20.4%) [42]. Additional data from Perinatal Services BC will be provided at the time of data analysis and data weighting.

### Participants

**Fig. 1:**
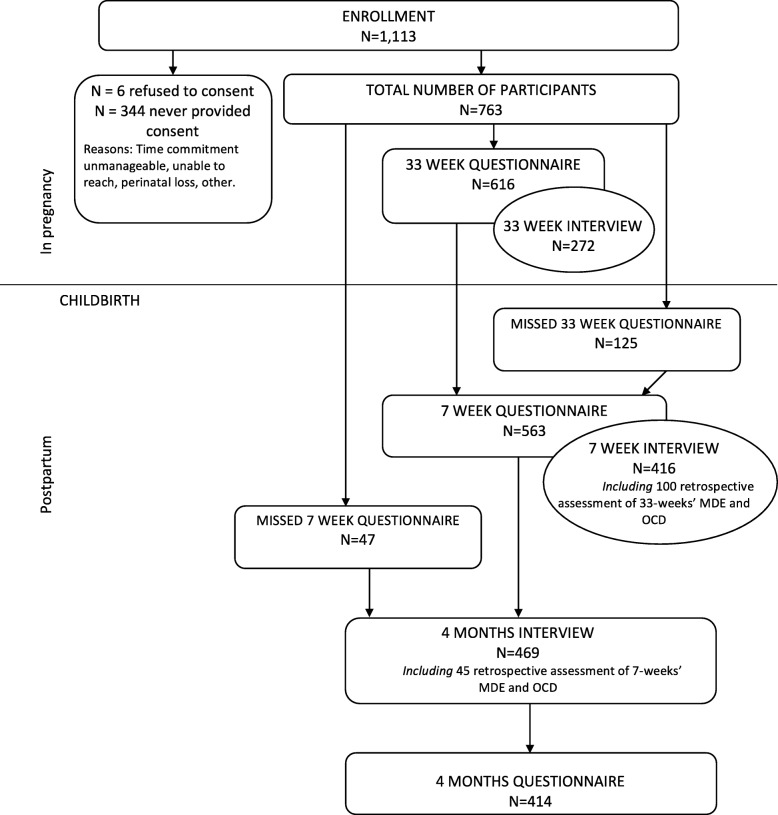
Participants’ flow through the study

Data were collected from February 09, 2014 until 14 February 2017. A total of 1113 women expressed interest in the study and were eligible to participate. A total of 763 women participated. Three hundred and six participants completed all three sets of questionnaires, and 170 completed all three interviews. In addition, 247 participants completed two full questionnaires and 237 participants completed two interviews (see Fig. 1).

The women who dropped out, did so because (a) they could not be reached, despite several attempts, (b) they were busy or working and no longer able to participate due to time constraints, or (c) they had at-risk pregnancies or concerns about their newborn’s health and no longer had the time and resources to participate.

Participants’ mean age was 32.36 years (SD = 4.92, min = 18.00, max = 46.75) at the time of the 33-week questionnaire. Most participants were married (74.1%) or living with a partner (20.2%). The remainder were single (4.2%), divorced (0.4%) or separated (1.1%). Many participants had attended an undergraduate program or college degree (52.3%; 13–16 years of education), or a postdoctoral program (36.6%; 17 years of education and above). Only 8.4% of participants did not continue their studies after completing their high school diploma and 2.6% did not complete high school. Participants were of European (52.7%), East Asian (11.5%), mixed (8.8%), South Asian (7.7%), Southeast Asian (6.2%), or Aboriginal (3.4%) heritage. Other ethnic heritages accounted for a small percentage of participants individually, but when combined, were endorsed by 9.6% of participants. The majority of participants were born in Canada (68.1%) and Asia (18.1%). Only 39.2% had never been pregnant before, and 56.9% had never given birth before. There were 689 singleton pregnancies, 31 twin pregnancies and one set of triplets.

Participants reported giving birth at the following locations: BC Women’s hospital (21.4%), Royal Columbian hospital (11.3%), Surrey Memorial hospital (11.2%), Victoria General hospital (8.7%), Saint Paul’s hospital (7.6%), Richmond hospital (6.9%), Burnaby General hospital (5.8%), Kelowna General hospital (5.6%), Abbotsford Regional hospital (5.4%), Lion’s Gate hospital (4.9%), Langley Memorial hospital (4.8%), at home (3.3%) or another location (3.1%). These numbers differ by 0.3 to 5.9% from the distribution of birth across hospitals in British Columbia, based on data provided by the British Columbia Perinatal Data Registry [42].

### Ethics

#### Ethics, consent and permissions

Ethics approval for this province-wide study was granted by the University of British Columbia Behavioural Research Ethics Board, the Vancouver Island Health Authority, the Vancouver Coastal Health Authority and the Fraser Health Authority. Due to the complexity of the study design and questions related to child abuse, we also consulted with the Ministry of Children and Family Development of British Columbia. They provided a letter of support, endorsing the study’s approach to confidentiality and the measures taken to protect participants. Initial written consent for the prenatal assessment (questionnaire and interview at 33 weeks) was obtained from participants via online and/or mailed forms at 33 weeks in pregnancy. Written consent for the post-partum assessments (both questionnaires and interviews at 7 weeks and 4 months) was obtained using a second consent form at 7 weeks postpartum. Oral consent was again given by participants at the beginning of each interview. Upon completion of their participation, participants received a letter explaining the current research and findings from previous studies.

#### Protection of participants and optimization of disclosure

The most challenging and unique aspects of this research were the legal-ethical elements. These were most apparent in two areas: (a) reports of unwanted, intrusive thoughts of infant-related *intentional* harm and (b) the assessment of socially unacceptable and potentially illegal parenting behaviours (i.e., physical and verbal aggression, and sexual behaviours toward the infant). We anticipated that many participants would feel threatened by both types of questions due to fear that by disclosing them, they would place themselves at risk (e.g., trigger a report to the Ministry of Children and Family Development, separation from their infant). In consideration of the above, we took several key steps to: (a) maximize open and honest disclosure by participants, and simultaneously (b) ensure the safety of participants. These steps are outlined below.

#### Thoughts of intentional harm

Based on various anecdotal reports of mothers whose infants were removed from their care because they disclosed unwanted, intrusive thoughts of intentionally harming their infant, our primary concern with respect to this portion of the research was that many women would be fearful of opening up about these kinds of thoughts. In an effort to optimize disclosure, we made it very clear to participants that: (a) unwanted, intrusive thoughts of infant-relate harm are a normative postpartum experience, (b) disclosure of such thoughts (no matter how horrific), in the absence of additional risk factors, would not result in any action taken by us, and (c) disclosure of a fear of acting on one’s postpartum harm thoughts, likewise, in the absence of any additional risk factors, would not result in any action taken by us. In summary, participants could safely disclose to us that: (a) they were experiencing unwanted, intrusive thoughts of harming their infant, and (b) they wear fearful of acting on these. This information was provided by interviewers and in the second consent form. Finally, in view of normalizing unwanted, intrusive thoughts, project interviewers, when introducing the harm thoughts interview, also disclosed their own experience of unwanted, intrusive thoughts.

#### Potentially reportable behaviours

There was a real risk that women might not respond honestly to questions about abusive or sexual behaviours. Further, disclosures of child harming behaviours could create a legal requirement of action on our part (i.e., a report to the Ministry of Children and Family Development). We took several steps to overcome barriers to disclosure and ensure participant safety while responding openly and honestly to these questions. Specifically, we asked about maternal aggression and sexual behaviour towards the infant in the final questionnaire package only, and when we asked about these behaviours we did so in such a way that made it impossible for us to link participants’ responses to their identity. This was especially important because this research involved numerous assessment points and therefore, it was not possible for the entirety of data collection to be anonymous.

We took all possible steps to ensure that once women had completed the final questionnaire in which reports of infant abuse may have occurred, and before we viewed these data, we: (a) severed all links between a woman’s data and her identity, (b) ensured in all respects that we would be unable to retrace our steps at a later time and determine the identity of any woman who disclosed child abuse. These steps were taken to ensure that, in addition, no outside agency could request or subpoena this information because the information no longer existed nor could be retraced.

The specific steps are outlined below.

#### Study codes

Each participant was assigned a randomly generated 10-digit alpha-numerical ID code. These study ID codes were created with a view to ensuring that team members would be unable to recall the ID codes of individual participants, thereby further ensuring that at the conclusion of participation, participant data be both anonymous and non-retraceable.

#### Data storage

Participants’ data were stored in five separate files:

1. A data tracking file with ID codes and information about progress through the study (e.g., interviews or surveys completed, completion dates and due dates) as well as general information such as the baby’s due date and date of birth.
2. Audio-files of participant interviews. These files contained no identifying information; ID codes only were used. In the event that a participant provided identifying information (e.g. her infant’s name) during one of the interviews, this information was edited out of the audio-file at the conclusion of the interview.
3. A data file containing ID codes along with responses to questionnaires and interviews.

These first two files, as well as the audio-files from the interviews, are stored on a secure server at the University of Victoria. The server was, and still is, accessed via VPN by research assistants if they have a valid University of Victoria’s email address and if they have been granted access by the principal investigator. During the data collection phase, access to those two data files was restricted to minimize errors and protect participants’ information.

4. A third file, called the *Master File*, contained the study ID codes and participants’ identifying information. It was stored on an encrypted USB key, which was stored in a locked briefcase. No electronic backup was made, in order to ensure the link between participants’ ID codes and identities existed in only one electronic document. A paper back-up copy was kept in the locked brief case. It was shredded and replaced each time the electronic file was updated. The encrypted key also allowed the data to be completely erased with no electronic ghost copy of the data. It ensured that neither our lab nor any outside agency would be able to later retrieve an earlier copy of this file. Over the course of the study, only essential personnel, including the principal investigator, had access to the encrypted key.
5. Paper-based packages and consent forms were stored in a locked filing cabinet.

#### Consent

The consent procedure was conducted at the point of recruitment and then repeated following childbirth to ensure that participants fully considered their interest in participating in the context of the emotional and life-changing experience of the birth of a child. The numerous protections to confidentiality, discussed above, were presented in detail only in the second (post-childbirth) consent form. Participants were informed of the reporting requirements under BC laws (i.e., a child in need of protection), and any other limits to confidentiality as well as the multiple steps taken to protect their safety and anonymity in the context of participation. It was our view that these issues needed to be raised with participants at the most relevant time, namely in the postpartum period where we ask question about intrusive, unwanted thoughts of infant-related harm and child abuse.

#### Email communication with participants

Participants were sent survey links by email and were contacted by phone or email to schedule interviews. In email communication, participants were addressed by their first names only. All email communication with participants was deleted once they were no longer needed.

#### Interviews

At the beginning of each interview, interviewers encouraged participants to share any questions and/or concerns they may have had about the interview process and participation in general. At the postnatal interview, limits to confidentiality were reviewed in detail with each participant before starting the interview, and any questions related to Consent #2 were answered. At the conclusion of each interview, women who reported mood or anxiety concerns were provided appropriate mental health referral information and/or referrals. Women who expressed concerns about their current level of support were called by the principal investigator to further discuss options in their community. For women who reported suicidal ideation and/or intent, level of risk was assessed (e.g., plan, lethality of the plan, means, etc.), and the principal investigator was contacted immediately. Appropriate steps were taken to ensure participant safety, including additional follow-up by the principal investigator.

#### Anonymization of data

When participants completed the final questionnaire or when they dropped out of the study, the link between their ID code and their name and other identifying information (a link which existed only on the encrypted USB key) was severed. Their ID codes were removed from the Master File permanently. The baby’s date of birth was deleted from the tracking file. In addition, when participants submitted their final questionnaire electronically, their email address and any identifying information was deleted from the survey provider’s address book. The survey provider confirmed with the study team that no records or back-ups would be kept. Although participants providing paper-based final questionnaires had been instructed to leave off all identifying information (e.g. name, return address) and include only their participant ID code and “final survey” on the envelope, these envelopes were nevertheless checked for the presence of any identifying information. If present, identifying information was permanently removed.

The purpose of the steps outlined above was to ensure that the link between participants’ identity and their participant ID and data was severed prior to opening 4-month questionnaires (which included questions about parenting practices and abuse). In addition, those questionnaires were not opened until a minimum of 20 other 4-month questionnaires were returned in the same manner (i.e. online surveys or paper questionnaires) from other participants. This procedure obscured which participant a particular questionnaire package belonged to.

The above procedure ensured that neither we, nor any outside agency would be able to later re-establish the link between participants’ data and their identities.

### Procedures

Women who expressed interest and met the study eligibility requirements were invited to participate. Participants were between 5 and 42-weeks gestation at the time of recruitment. At 33-weeks in pregnancy (or immediately following recruitment if they joined the study after 33-weeks) and at approximately 5 to 7-weeks postpartum, women completed an online questionnaire, followed by an interview. At 3–4-months postpartum, participants completed an interview followed by an online questionnaire (see Table 1). Participants who missed the 33-week or the 7-week questionnaires were asked to complete a short online questionnaire (i.e. missed 33-weeks or missed 7-weeks questionnaires) for the purpose of collecting demographic and birth information. Any participant who could not easily access the online questionnaires was mailed a paper copy of the questionnaires to be returned by mail.

**Table 1:** Summary of measures

Domain	Measure	Method	Administrator	Location
Data collection at 33-week in pregnancy				
Consent 1	Written consent Oral consent	Consent form Check-in by interviewer	Participant Interviewer	Online Phone
Characteristics	Demographic and reproductive history information	Self-report questionnaire	Participant	Online
Symptoms	Edinburgh Postnatal Depression Scale (EPDS) Obsessional Beliefs Questionnaire (OBQ-44) Dimensional Obsessive-Compulsive Scale (DOCS)	Self-report questionnaires	Participant	Online
Other areas of functioning	Medical Outcome Study Social Support Survey (MOS-SSS) Pittsburg Sleep Quality Index (PSQI) Relationship Styles Questionnaire (RSQ)	Self-report questionnaires	Participant	Online
Diagnosis (OCD, MDE)	Structured Clinical Interview for DSM-IV (modified to reflect the DSM-5)	Structured Interview	Interviewer	Phone
Data collection at 7-week post-partum				
Consent 2	Written consent Oral consent	Consent form Check-in by interviewer	Participant Interviewer	Online Phone
Characteristics	Birth Information	Self-report questionnaire	Participant	Online
Symptoms	Edinburgh Postnatal Depression Scale (EPDS) Obsessional Beliefs Questionnaire (OBQ-44) Dimensional Obsessive-Compulsive Scale (DOCS) Interpretations of Intrusions Inventory (III)	Self-report questionnaires	Participant	Online
Other areas of functioning	Medical Outcome Study Social Support Survey (MOS-SSS) Pittsburg Sleep Quality Index (PSQI) Child Abuse Potential Inventory (CAP Inventory)	Self-report questionnaires	Participant	Online
Diagnosis (OCD, MDE)	Structured Clinical Interview for DSM-IV (modified to reflect the DSM-5)	Structured Interview	Interviewer	Phone
Thoughts of harm related to the baby	Post-Partum Intrusions Interview (PPII) Yale-Brown Obsessive Compulsive Scale (Y-BOSC)	Structured Interview	Interviewer	Phone
Data collection at 4-month post-partum				
Consent	Oral Consent	Check-in by interviewer	Interviewer	Phone
Symptoms	Edinburgh Postnatal Depression Scale (EPDS) Obsessional Beliefs Questionnaire (OBQ-44) Dimensional Obsessive-Compulsive Scale (DOCS) Interpretations of Intrusions Inventory (III)	Self-report questionnaires	Participant	Online
Other areas of functioning	Medical Outcome Study Social Support Survey (MOS-SSS) Pittsburg Sleep Quality Index (PSQI) Parenting Behaviours Questionnaire (PBQ)	Self-report questionnaires	Participant	Online
Diagnosis (OCD, MDE)	Structured Clinical Interview for DSM-IV (modified to reflect the DSM-5)	Structured Interview	Interviewer	Phone
Thoughts of harm related to the baby	Post-Partum Intrusions Interview (PPII) Yale-Brown Obsessive Compulsive Scale (Y-BOCS)	Structured Interview	Interviewer	Phone

#### Interviews

Interviews were conducted over the phone, at each assessment point.

### Assessment tools

#### Diagnostic instrument

The *Structured Clinical Interview for DSM-IV (SCID-IV)* [43] is a well-validated structured diagnostic interview. The SCID-IV was used to assess OCD and MDE, although the wording was adapted to be consistent with DSM-5. OCD diagnosis was the primary focus of the present study and participants’ diagnostic status was categorized as follows: absent, sub-clinical, full criteria. If a participant had met full criteria at an earlier assessment point, then two other categories, in partial remission or in full remission, were options. MDE diagnostic status was coded as absent or full criteria.

Symptom severity was rated on a scale of 0 (none) to 8 (very severe/disabling). Symptom severity ratings were assigned to the diagnoses as follows: 0 = Absent or In Full Remission; 0.5 to 2.5 = In Partial Remission; 3 to 3.5 = Sub-Clinical; 4 to 8 = Full Criteria.

With regards to the OCD diagnostic assessment, additional questions were included to elicit obsessions relating to accidental or intentional infant-related harm. After enquiring about usual obsessive-compulsive thoughts using the modified SCID-IV, interviewers reminded participants of any thoughts of infant-related harm that they had endorsed on the Postpartum Intrusions Interview (see below), and then enquired about the frequency and repetitiveness of those thoughts. In order to be considered as obsessions (relevant to a diagnosis of OCD), thoughts of infant-related harm were required to be repetitive, unwanted and intrusive. In addition, interviewers clarified inconsistencies between participants’ reports in the modified SCID-IV OCD section and in the Postpartum Intrusions Interview. For example, if a participant felt that her thoughts of infant-related harm were not repetitive but had previously endorsed the thoughts occupying 3 h per day, the interviewer would attempt to clarify the participant’s self-report.

#### Postpartum thoughts of harm

Maternal postpartum intrusive and unwanted thoughts of accidental and intentional harm were assessed using the *Postpartum Intrusions Interview (PPII)* [1]*,* an instrument developed by the PI in prior research on this topic*.* Items assessed mothers’ intrusive, unwanted thoughts of accidental and intentional harm to the infant, as well as behavioural responses to the thoughts (e.g., avoidance of being alone with the baby). The interviewers asked participants to identify when the thoughts first started, when they became most intense and if they were still occurring. To promote frank disclosure, the interview began with normalizing information that thoughts of this kind are common amongst new mothers. To further establish rapport and comfort, the interviewer provided examples of intrusive, unwanted thoughts of harm commonly reported by new mothers in relation to the newborn. Whenever possible, interviewers provided examples of their own, intrusive, unwanted postpartum thoughts of infant-related harm.

#### Yale-Brown obsessive compulsive scale (Y-BOCS) [44]

The Y-BOCS is an interviewer-rated scale that measures the severity of obsessions and compulsions on a 0–4 scale [44]. The Y-BOCS was administered separately to assess for the frequency and severity of unwanted and intrusive thoughts, identified by the PPII, of (a) accidental harm, and (b) intentional harm related to the infant, and their related compulsions. Participants were asked to report on the week prior to the interview and the most intense week postpartum (7-week interview) or since the last interview (4-months interview). The Y-BOCS has demonstrated adequate to excellent internal, test-retest and interrater reliability [45, 46], as well as good criterion-related and convergent validity [45]. However, a recent meta-analysis showed statistically significant heterogeneity in reliability estimates across studies. This heterogeneity seemed in part due to variations in standard deviation and mean of test scores, the nature of the sample (non-clinical samples had higher internal reliability estimates), and participants’ disorder history (with longer history being related to lower internal reliability estimates) [46].

### Training

There were a total of 10 interviewers, including the principal investigator. Interviewers had, at a minimum, completed an undergraduate degree in psychology or a related discipline, and most were enrolled in a counselling or clinical psychology graduate degree program at the University of British Columbia or the University of Victoria. All interviewers were trained to administer the PPII and SCID-IV by the principal investigator or a senior interviewer. All interviewers were required to administer interviews under direct observation by the principal investigator until she was satisfied that the trainee was competent to conduct interviews independently. For the PPII, interviewers had to demonstrate their capability in explaining the difference between worry thoughts and intrusive thoughts to participants, and to know when and how to seek clarity when participants provided unclear or inconsistent responses. In order to be deemed competent to administer the SCID-IV, interviewers were required to match the principal investigator on the OCD and MDE diagnoses, within one severity point of the severity rating given by the principal investigator, on a minimum of two successive interviews.

As part of their training, interviewers were also made aware of some of the specific issues relevant to interviewing new mothers. For example, interviewers were trained to differentiate between symptoms of depression and obsessive-compulsive disorder, and normal changes due to pregnancy or motherhood (e.g., loss of energy, appetite, sleep and difficulty concentrating). Interviewers were supervised by the principal investigator via face to face and phone contact, with listening to audio-tapes of interviews as necessary.

### Reliability checks

Upon completion of the study, reliability checks were completed by a senior interviewer and OCD specialists external to the team. Of the interviews with an audio-file, 25 % with significant OCD symptomatology (sub-clinical, clinical, partial remission) were selected, whereas 5 % with no diagnosis of OCD were selected. Interviews were also proportionally and randomly sampled from each interviewer and at each time point.

#### Measures

Questionnaires administered to participants are described below.

##### Demographic, reproductive history, pregnancy and infant health information

This information was collected via self-report. We asked for demographic information (i.e., age, marital status, occupation, education, income, race/ethnicity, and language), pregnancy information (i.e., medical and pregnancy complications, and reproductive history), and birth information (i.e., baby’s date of birth, mode and location of delivery, birth weight, pregnancy and birth complications, neonatal health, and infant feeding).

##### Dimensional obsessive-compulsive Scale [47] (DOCS)

The DOCS is a 20-item self-report measure [47]. The factor structure of the DOCS indicates a 4-factor model corresponding to the four most consistently replicated OCD symptom dimensions and the measure’s subscales: a) germs and contamination, b) responsibility for harm, injury or bad luck, c) unacceptable obsessional thoughts and d) symmetry, completeness and exactness [47–49]. Within each symptom dimension, five items (rated 0 to 4) assess the parameters of severity. DOCS subscales have excellent reliability in clinical samples (a = .87–.96) and in student samples (a = .82–.93) as well as convergent, discriminant and construct validity [47–49]. The measure is sensitive to changes over time [47–49] and incremental increases on the DOCS represent actual increases in OCD symptoms [48].

##### Edinburgh postnatal depression Scale [50] (EDPS)

The EPDS is a 10-item self-report measure designed to screen for postnatal depression. It has demonstrated good to excellent psychometric properties in 11 countries [51]. The sensitivity and specificity of the EPDS are in acceptable ranges (70–100% for sensitivity and 74–97% for specificity in the antenatal period; 65–100% for sensitivity and 49–100% for specificity in the postnatal period) [51, 52]. Evidence suggests the measure’s cut-offs might vary at different time points in pregnancy and postpartum and in different cultures [51]. The EPDS is a widely used screening tool for postpartum depression [53].

##### Obsessional beliefs Questionnaire [54] (OBQ-44)

The OBQ-44 is a 44-item self-report measure designed to measure attitudes relevant to intrusive thoughts including: responsibility and threat estimation, perfectionism and need for certainty, and importance of control. The 44 items represent these 3 belief domains and are independent of OCD symptoms. Psychometric properties of the OBQ are considered good [54].

##### Interpretations of intrusions Inventory [54] (III)

The III is a 31-item self-report measure of interpretations of the respondent’s recent intrusive thoughts, images, and impulses, representing the domains from the OBQ-44 (see above). Items are scored from 0 to 10 and are consistent with theory and research concerning appraisals of intrusive thoughts.

##### Child abuse potential inventory [55] (CAP Inventory)

The CAP Inventory is a 160-item self-report measure of parental attitudes and beliefs. Items are scored as Agree or Disagree. The CAP contains six parenting sub-scales, three validity scales and a response distortion index. The CAP possesses excellent psychometric properties and cross-cultural validity [55–57]. In a recent comprehensive review, the CAP was one of the only parenting skills and attitude measures (out of 25 measures) with evidence for at least adequate internal, test-retest, and cross-informant reliability as well as content, predictive, convergent, and discriminant validity [57].

##### Medical outcomes study social support Survey [58] (MOS-SSS)

The MOS-SSS is a 19-item self-report measure of social support. Items assess five dimensions of social support: functional, tangible, affectionate, positive social interaction and emotional/informational. Respondents estimate how often different types of social support are available to them on a 1 to 5 scale [58]. This measure has demonstrated good internal consistency, test-retest reliability, construct validity and a stable factor structure in clinical and non-clinical populations [58, 59].

##### Pittsburgh sleep quality Index [60] (PSQI)

The PSQI [60] is a 19-items self-report measure of sleep quality and disturbance over the past month. Higher scores are indicative of reduced sleep quality. The scale has shown good internal consistency and test–retest reliability, as well as good construct validity across several studies [60–63], and when administered to pregnant women [64]. The PSQI demonstrated moderate structural validity [63], and a two-factor model when administered to pregnant women [64]. It is the most commonly used sleep measure in clinical and research settings [63].

##### Relationship styles Questionnaire [65] (RSQ)

The RSQ is a 30-item self-report measure of attachment in close relationships. It assesses one’s working models of self and others and the person’s relative fit to four theoretical attachment styles: secure, fearful, preoccupied and dismissive [65]. Its subscales exhibit adequate to good internal consistency and show correlations with relationship satisfaction in the expected directions [65–67]. The RSQ can be divided into its original subscales [65–67] or into two-factors: a Secure-Anxious attachment dimension and a Secure-Avoidant attachment dimension [68].

##### Parenting Behaviours questionnaire (PBQ)

The PBQ is a 13-item self-report questionnaire assessing verbal, physical and sexual abuse of the infant. Participants who endorsed engaging in verbally, physically and/or sexually abusive acts were also prompted to indicate whether they did so to cope with an intrusive thought. While the Conflict Tactics Scale-Parent Child version has been widely used in the literature as a measure of child abuse [69], several of its items are not applicable to young mothers and their infants. For example, items such as “you put your child in time out (or sent the child to his or her room” or “you took away privileges or grounded your child” would only be appropriate for children out of infancy [69]. Consequently, the study team, which included one expert in the area of child abuse, developed the PBQ for the present study. The PBQ directly asks, in a face-valid manner, a series of questions about various aggressive behaviors participants may have engaged in with their infants. For example, items include: “you shook your baby” or “you screamed or yelled at your baby”. Participants are asked if they have engaged in any of these behaviors at any time since their baby’s birth, and how often they have engaged in each behavior using a scale ranging from never to 3+ times.

### Current status

Data collection for the study is complete. A substantial portion of the data set has been cleaned and the reliability checks for OCD diagnoses have been completed. Reliability checks for MDE diagnoses have not yet been conducted. Data analyses and manuscript preparation are now currently underway.

### Data analysis plan

Data analysis will be conducted using SPSS, Version 24 and R version 3.5.0.

#### Descriptive statistics

Descriptive information will be presented in the form of means, standard deviations, proportions, percentages and 95% confidence intervals.

#### Missing data

Participants with missing data at one or more time points will be included in the analyses, as long as missing data are ignorable. Ignorable missing data are those which can be considered missing completely at random (e.g., participants who move or change phone numbers), or missing at random (missing data predictable from scores at previous time points, but not related to the values of the missing data themselves).

#### Specific analyses

##### Perinatal intrusive thoughts of infant-related harm

a) The prevalence of maternal intrusive and unwanted thoughts of infant-related harm at 7-weeks and 4-months postpartum will be presented in the form of percentages with 95% confidence intervals.

b) Generalized mixed effects models for longitudinal data will be used to assess the course of the frequency, intensity, and evoked distress of unwanted intrusive thoughts of infant-related harm over the first 4-months postpartum.

c) The content and characteristics of new mothers’ unwanted, intrusive thoughts of infant-related harm will be presented descriptively in text and tables, as well as in the form of means and standard deviations (e.g., for distress associated with the thoughts), as well as percentages with 95% confidence intervals (e.g., thoughts of suffocation). d) Multiple logistic regression will be used to assess potential predictors of postpartum OCD symptoms. Specific predictors will include, among others, beliefs about unwanted, intrusive thoughts of infant-related harm and the frequency and duration of those thoughts, depressive symptoms (in pregnancy and postpartum), history of OCD symptoms, social support, and attachment.

##### Maternal aggression toward the infant

a) Chi-square analysis will be used to test for a possible difference in the prevalence of child harming behaviours among women who report unwanted, intrusive, postpartum thoughts of intentional harm related to their infant, compared with women who do not report infant-related thoughts of intentional harm.

b) Multiple logistic regression will be used to assess potential predictors of maternal aggression towards the infant. Specific predictors will include, among others, parenting beliefs, maternal sleep, social support, negative mood, symptoms of OCD and unwanted, intrusive, infant-related thoughts of intentional harm.

#### Perinatal OCD and MDE

Perinatal OCD and MDE point and period prevalence and incidence (postpartum only) data will reported with proportions and 95% confidence intervals as well as Poisson regression. The latter will allow us to calculate the incidence rate of new diagnoses post partum, adjusted for person-time of follow up. Person time will be calculated as the time (in weeks) post-partum at which the onset of OCD and MDE occurred, or censored at the time of the last contact for those without OCD and MDE. The incidence rates between groups of women (e.g. those with intrusive thoughts vs not, high vs low social support) will be compared using Poisson regression models with person-time as an offset.

A secondary objective of this research was to determine the prevalence and course of perinatal MDE:

- The prevalence of MDE at 7-weeks and 4-months postpartum will be presented in the form of percentages with 95% confidence intervals.
- Generalized mixed effects models for longitudinal data or survival analysis will be used to assess the trajectory of MDE and MDE symptoms over the prenatal and postnatal period.

## Discussion

Infant-related accidental and intentional harm thoughts, that are unwanted and intrusive, have received very limited attention in the empirical and clinical literature [4]. Our longitudinal study aims to address the wide gaps in the literature-base and provide much-needed guidance for the care providers, child protection workers and policy makers responding to the sensitive situations in which a mother discloses unwanted and intrusive thoughts of accidental and/or intentional harm related to their infant.

Previous evidence suggests that unwanted, intrusive thoughts of infant-related harm are not associated with an increased risk of actual harm to the infant [1, 4]. However, this finding is based on a paucity of studies which were reliant on relatively small sample sizes. It is therefore unsurprising that the finding has not prompted a shift in current clinical or research practices. Additional evidence is required in order for this finding to influence the risk appraisal that follows a mother’s disclosure of intrusive and unwanted thoughts of infant-related harm. Our primary goal is to provide this evidence, determining the nature and degree (if any) of association between maternal unwanted, intrusive thoughts of infant-related harm and harm toward the infant.

Gaps within the evidence base exist beyond this association, and unanswered questions remain concerning the course and prevalence of postpartum intrusive thoughts of infant-related harm, their association with depression and obsessive-compulsive symptoms, and the specific risks that these thoughts pose to the well-being of new mothers and their infants. These areas of interest are of particular importance given the impact that these disorders can have. Furthermore, women suffering from anxiety disorders such as OCD are more likely to suffer from depression [27], and depressive symptoms can slow down the resolution of obsessive-compulsive symptoms in the perinatal period [28]. Thus, our additional goals are to a) assess the course of depression and OCD in the perinatal period and their co-morbidity, and b) build on the existing literature concerning risk factors for depression and OCD in the perinatal period.

This study incorporated many new and informative elements. First and foremost, we are, to our knowledge, the first research team to assess the relationship between unwanted and intrusive thoughts of infant-related harm and maternal harming behaviors toward the infant. Secondly, we developed complex and comprehensive procedures to guarantee the protection of participants and our approach to ethics may provide ideas or guidelines for other researchers needing to anonymize harming behavior data in the context of longitudinal studies. Our procedures were innovative and received support from the BC Ministry of Children and Families. In addition, we believe our efforts to ensure confidentiality and anonymity likely led to greater disclosure and thus more accurate reporting. Thirdly, participants were recruited proportionally across multiple hospitals to maximize the likelihood of a representative sample.

Despite a recruitment method designed to optimize the representativeness of our sample, a higher attrition rate was observed than anticipated across all three time points. While the loss of participants reduced our sample size to a reasonable but less than ideal number, we were nevertheless able to achieve a sample size sufficient to provide informative data and answers to our key research questions. As participation involved lengthy and extensive assessments, this presented a barrier for some new mothers. In addition, new mothers self-selected to participate. Consequently, our sample may not be fully representative of the population from which it was drawn. It is possible that new mothers who completed this study were, on average, higher functioning, had more social support and resources, or differed in other important ways from the new mothers who declined to participate or dropped out. Where possible, we will test for differences between dropouts and completers or compare our completers with statistics provided by British Columbia Perinatal Data Registry.

Finally, we used gold standard procedures for the assessment of obsessive-compulsive disorder and depression. In our assessment of obsessive-compulsive symptoms, we included unwanted, intrusive thoughts of infant-related harm, as the content of obsessive thoughts is often related to one’s on-going concerns. Including those thoughts might allow us to provide a more accurate estimate of the prevalence of obsessive-compulsive disorder in the perinatal period. Less interviews were completed in pregnancy than in the postpartum period. Consequently, a portion of the diagnoses of OCD and MDE in pregnancy are based on retrospective reports. This limitation will be partially addressed by triangulating participants’ retrospective self-report with their responses to self-report measures administered in pregnancy, in the cases of participants who completed the full 33-weeks questionnaire.

Findings from this study have the potential to increase (a) our understanding of obsessive-compulsive and depressive symptoms in the perinatal period, and (b) our knowledge about unwanted, intrusive thoughts of infant-related harm, frequently experienced by new mothers and their correlates. Our goal will be to translate our findings into clear guidelines for care providers working with new mothers, and to improve assessment and treatment of obsessive-compulsive and depressive symptoms in the perinatal period.

## Abbreviations

BC: British Columbia
CAP Inventory: Child Abuse Potential Inventory
DOCS: Dimensional Obsessive-Compulsive Scale
DSM-5: Diagnostic and Statistical Manual of mental disorders-5
EPDS: Edinburgh Postnatal Depression Scale
III: Interpretations of Intrusions Inventory
MDE: Major Depressive Episode
MOS-SSS: Medical Outcomes Study Social Support Study
OBQ-44: Obsessive Beliefs Questionnaire
OCD: Obsessive-Compulsive Disorder
PBQ: Parenting Behaviours Questionnaire
PPII: Postpartum Intrusion Interview
PSQI: Pittsburgh Sleep Quality Index
RSQ: Relationship Style Questionnaire
SCID-IV: Structural Clinical Interview for DSM-IV
Y-BOCS: Yale-Brown Obsessive Compulsive Scale
